# Dynamic biclustering of microarray data by multi-objective immune optimization

**DOI:** 10.1186/1471-2164-12-S2-S11

**Published:** 2011-07-27

**Authors:** Junwan Liu, Zhoujun Li, Xiaohua Hu, Yiming Chen, EK Park

**Affiliations:** 1 School of Computer and Information Engineering, Central South University of Forestry and Technology, Changsha 410004, China; 2 State Key Laboratory of Software Development Environment, Beihang University, Beijing 100191, China; 3 Beijing Key Laboratory of Network Technology, BeiHang University, Beijing 100191, China; 4 College of Information Science and Technology, Drexel University, Philadelphia, PA 19104, USA; 5 School of Information Science and Technology, Hunan Agricultural University, Changsha 410128, China; 6 CSI-CUNY in Staten Island, NY, USA

## Abstract

**Abstract:**

**Background**

Newly microarray technologies yield large-scale datasets. The microarray datasets are usually presented in 2D matrices, where rows represent genes and columns represent experimental conditions. Systematic analysis of those datasets provides the increasing amount of information, which is urgently needed in the post-genomic era. Biclustering, which is a technique developed to allow simultaneous clustering of rows and columns of a dataset, might be useful to extract more accurate information from those datasets. Biclustering requires the optimization of two conflicting objectives (residue and volume), and a multi-objective artificial immune system capable of performing a multi-population search. As a heuristic search technique, artificial immune systems (AISs) can be considered a new computational paradigm inspired by the immunological system of vertebrates and designed to solve a wide range of optimization problems. During biclustering several objectives in conflict with each other have to be optimized simultaneously, so multi-objective optimization model is suitable for solving biclustering problem.

**Results**

Based on dynamic population, this paper proposes a novel dynamic multi-objective immune optimization biclustering (DMOIOB) algorithm to mine coherent patterns from microarray data. Experimental results on two common and public datasets of gene expression profiles show that our approach can effectively find significant localized structures related to sets of genes that show consistent expression patterns across subsets of experimental conditions. The mined patterns present a significant biological relevance in terms of related biological processes, components and molecular functions in a species-independent manner.

**Conclusions**

The proposed DMOIOB algorithm is an efficient tool to analyze large microarray datasets. It achieves a good diversity and rapid convergence.

## Background

Rapid development of the DNA microarray technology makes it very possible to study the transcriptional response of a complete genome to different experimental conditions. The rapid increasing of microarray datasets provides unique opportunities to perform systematic functional analysis in genome research. A subset of genes showing correlated co-expression patterns across a subset of conditions are expected to be functionally related. One important research area in bioinformatics and clinical research is finding patterns which relate to disease diagnosis, drug discovery and the function prediction.

Biclustering is proposed for grouping simultaneously genes set and condition set over which the gene subset exhibit similar expression patterns. Cheng and Church [1] introduce first biclustering to mine genes clusters with respect to a subset of the conditions from microarray data. Up to date, a number of biclustering algorithms for microarray data analysis have been developed such as δ-biclustering [1], FLOC [2], pClustering [3], statistical-algorithmic method for biclustering analysis(SAMBA) [4], spectral biclustering [5].

During the last three decades, inspired by biology views, some heuristic approachs such as evolutionary algorithms [6] have been proposed to discover global optimal solutions in gene expression data. For multi-objective optimization (MOO) problem, multi-objective evolutionary algorithms (MOEAs) [7,8] are proposed to discover efficiently global optimal solution.

Recently an artificial immune system is introduced to deal with MOO problem. Jiao [9] proposes immune genetic algorithm(IGA) which improves the searching ability and adaptability, greatly increase the converging speed. Yoo and Hajela [10] first extends the immune system to solve multi-objective optimization problems. Coello [11,12] propose an algorithm based on the immune response principle to solve MOO problem and effectively improve the diversity of Pareto optimal solutions. BIC-aiNet (Artificial immune Network for Biclustering) [13] being an immune-inspired biclustering algorithm is used to group similar texts efficiently and extract implicit useful information from groups of texts. Coelho [14] combines the multi-population of aiNet and the biclustering techniques, and proposes MOM-aiNet (Multi-Objective Multi-population Artificial Immune Network) algorithm to mining biclusters. Liu[15] proposes a novel multi-objective immune biclustering (MOIB) algorithm to find more significant biclusters from gene expression data.

Most MOPs use a fixed population size to find non-dominated solutions for obtaining the Paterto front. The computational cost is the greatest influence of population size on these population-based meta-heuristic algorithms. Hence dynamically adjusting the population size need consider the balance between computational cost and the algorithm performance. Some methods using dynamic size are proposed. Tan [16] proposed an incrementing MOEA(IMOEA) that adaptively computes am appropriate population size according to the online discovered trade-off surface and its desired population size that corresponds to the distribution density. Yen and Lu [17] proposed dynamic population size MOEA(DMOEA) that includes a population-growing strategy based on the converted fitness and a population-declining strategy that resorts to the following age, health and crowdedness. Leong and Yen [18] introduced dynamic population size and a fixed number of multiple swarms into multi-objective optimization algorithm that improved diversity and convergence of optimization algorithm.

## Methods

Based on the immune response principle and ε-dominance strategy [19], this paper incorporating dynamic population size [18] into MOIB [15] algorithm, and proposes a novel dynamic multi-objective immune optimization biclustering(DMOIOB) algorithm to find one or more significant biclusters of maximum size in microarray data. In the proposed algorithm, the feasible solutions are regarded as antibodies and Pareto optimal solutions are preserved in an antigen population updated by ε-dominance relation and computation of crowding distance. Many Pareto optimal solutions can be effectively obtained and distributed onto the Pareto front in this way. Three objectives, the size, homogeneity and row variance of biclusters, are satisfied simultaneously by applying three fitness function in optimization framework. A low mean squared residue (MSR) score of bicluster denotes that the expression level of each gene within the bicluster is similar over the range of conditions. Therefore, we focus on finding biclusters of maximum size, with mean squared residue lower than a given δ, with a relatively high row variance.

### Biclusters

Given a gene expression data matrix D=G×C={*d_ij_*} (here *i*∊[1, *n*] , *j*∊[1, *m*]) is a real-valued *n*×*m* matrix, here G is a set of *n* genes {g_1_, g_2_, ⋯, g_n_}, C a set of m biological conditions {c_1_, c_2_, ⋯, c_n_}. Entry *d_ij_* means the expression level of gene *g_i_* under condition *c_j_.* If there is a submatrix B=*g*×*c*, where *g*⊂G, *c*⊂C, to satisfy certain homogeneity and minimal size of the cluster, we say that B is a bicluster.

### Bicluster encoding

Each bicluster is encoded as an individual of the population. Each individual is represented by a binary string of fixed length *n*+*m*, where *n*, *m* is the number of genes, conditions of the microarray dataset, respectively. The first n bits are responding to n genes, the following m bits to m conditions. If a bit is set to 1, it means that the responding gene or condition belongs to the encoded bicluster; otherwise it does not. This encoding presents the advantage of having a fixed size, thus using simply of standard variation operations. Therefore, the string “0110100010#0110100110” presents the individual encoding a bicluster with 4 genes and 5 conditions, and its size is 4×5=20. Where # is a symbol used to delimit the bits of the rows from the columns.

### Fitness function

Our hope is mining biclusters with low mean squared residue, with high volume and gene-dimensional variance, and those three objectives in conflict with each other are well suited for multi-objective to model. To achieve these aims, this paper uses the same fitness functions as [20].

### Update of ϵ-Pareto set of the population

In order to guarantee the convergence and maintain diversity in the population at the same time, we implement updating of ϵ-Pareto set of the population during clonal selection operation. A general scheme of the updating algorithm is given in [19].

### Immune response principle

An immune system can collect biological processes of an organism that protects against disease by identifying and killing pathogens and tumour cells. It can detect a wide variety of viruses and parasitic worms, and distinguish them from the organism's own healthy cells and tissues to protect an organism. It is highly distributed, highly adaptive, self-organization in nature [21]. Artificial Immune System (AIS) is a new computational approach for the computational intelligence community. It has widely such as pattern recognition, data analysis, function approximation and optimization.

The immune selection principle [22] is used to describe the basic properties of an adaptive immune response to an antigenic stimulus [21]. When applying the immune selection principle to solve multi-objective problem, it can generate several elements from the Pareto optimal set at one run. Clonal selection operation is used to implement local search in many different directions along the Pareto front. Mutation operator is applied to explore through the whole search space, thus attain the exact Pareto front of the problem.

### DMOIO biclustering algorithm

Multiple-objective optimization aim at the following two competing objectives: 1) to quickly obtain a non-dominated front that is close to the true Pareto front and 2) to maintain the diversity of the solutions along the resulting Pareto front. These two objectives are in conflict each other because maintaining the diversity will slow down the convergence speed and may degrade the quality of the resulting Pareto front. On one hand, MOIO algorithms tend to the optimal regions. On the other hand, the clonal selection behaviour may lead to premature convergence in the search space and produce a uniformly distributed Pareto front. The influence of population size on the performance of MOIO is the computational cost. It is difficult to deal with this conflict issues for a MOIO with a fixed population size because a predetermined computation resource has to be allocated and properly distributed between two competing objectives. Hence, inspired by [18], during biclustering of the microarray datasets, dynamically adjusting the population size to explore the search space in balance between two competing objectives is applied in this paper.

### Initial population

In most multi-objective optimization methods the initial archive is set to empty. The first archive contains the non-dominated solutions of the initial population. Each antigen selects best local guide from the archive members using Sigma method [23]. Selecting the first local guides from the archive has a great impact on the diversity of solutions in the next generations. Hence the diversity of solutions depends on the first non-dominated solutions. But if the initial archive is not empty and contains some well-distributed non-dominated solutions, the solutions converge faster than before, while keeping a good diversity. There are two methods to find a good initial archive. The first possibility is to run the MOIO with an empty archive for a large population and a few generations. The large population gives us a good diversity and a few generations (e.g., 10 generations) are used to develop the population to a little convergence. On another hand, MOEA can produce some good solutions with a very good diversity after a few generations. So another possibility is to use the results of a small MOEA method. Here, small means a MOEA with a few individuals and a few generations (e.g., 10 individuals and 10 generations). This paper first runs state-of-art MOEA(NSGA-II [24] ) with 30 individuals and 10 generations to produce the initial archive of DMOIOB.

### Fining the global best solution

To order to find the global best solutions, this paper uses the basic idea of Sigma method [23] and by considering the objective space, finding the best local guide p_g_ among the archive members for the antigen ***i***of population as follows. In the first step, we assign the value σ_j_, to each antigen *j* in the archive. In the second step, σ_i_for antigen ***i*** of the population is calculated, and then calculates the distance between the σ_i_ and σ_j_**, **∀_j_*j*=*1*,*⋯*,*|A|*. Finally, the antigen kin the archive A which its σ_k_has the minimum distance to σ_i_ is selected as the best local guide for the antigen i. Therefore, antigen p_g_ = x_k_is the best local guide for antigen i.In other words, each antigen that has a closer sigma value to the sigma value of the archive member, must select that archive member as the best local guide. In the case of two dimensional objective spaces, closer means the difference between the sigma values and in the case of m- dimensional objective space, it means the m-dimensional Euclidean distance between the sigma values. Algorithm 1 shows the algorithm of the Sigma method for finding the best local p_g_ for the antigen i of the population [23]. Here, the function Sigma calculates the σ value and dist computes the Euclidian distance. y_i_ denotes the objective value of the jth element of the antigen population *P*.

### Population adding method

Population adding strategy mainly consist in increasing the population size to ensure sufficient number of individuals to contribute to the search process and to place those new individuals in unexplored areas to discover new possible solutions. Based on the strategies of dynamic population size [18], the following procedures is proposed to facilitate exploration and exploitation capabilities for DMOIOB.

**Step 1:** Selecting candidate antibodies added

The non-dominated set considered as candidate antibodies must have the highest probability of generating new antibodies that will improve the convergence toward the Pareto front. Therefore the number of potential antibodies determined via ns = INT(r1× (total no. of antibodies in non-dominated set)) is randomly selected from the non-dominated set. Where *r_1_* denotes a random number obtained from a uniform distribution within [0, 1].

**Step 2:** Defining the number of mutation

The number of mutation of the selected antibody is adaptively determined every iteration. Each selected antibody’s responsibility is to generate a certain number of new antibodies from the selected antibody. A probability value is used to determine the number of perturbations adaptively in which the number of mutation (number of new antibodies to be generated) is bound by the minimum and maximum number of mutation.

**Step 3:** Limiting the range of new antibodies

In proposed algorithm, to balance the exploitation and exploration capabilities and to avoid generating too many new antibodies from being too far away from the selected antibodies, it is necessary to generate a higher number of new antibodies within the neighbourhood than outside of the neighbourhood which similar to [16].

### Population decreasing method

To prevent the excessive growth in population, a population decreasing strategy which similar to [16] is proposed to adaptively control the population size. In DMOIOB, the condition to remove a antibody depends upon Sigma values. Sigma value is utilized to select potential antibodies to be deleted. After computing all the distance between Sigma value of each antibody and Sigma value of its corresponding best local guide, the rank of the distance of each antibody can be attained. If the removal of antibodies is only based upon the distance rank of each antibody, then there is a possibility of eliminating an excessively large quantity of antibodies in which some may carry unique schema to contribute in the search process. A selection ratio is implemented to regulate the number of antibodies to be removed and to provide some degrees of diversity preservation at the same time. A selection ratio that is inspired by Coello and Montes [25] is used to stochastically allocate a small percentage of antibodies in the population for removal. Hence, given a selection ratio *S* ∊ [0, 1], at iteration *t*, the number of antibodies to be eliminated is *S*×|Pt|. Note that the choice of the selection ratio is dependent upon the user’s preference, where it can be a function of the swarm population size or it can be a fixed ratio. For this paper, the selection ratio is a fixed number, which is set to be a small number, i.e., *S* ≤ 0*.*2. With a small selection ratio, there is a possibility that those selected antibodies in *Pt* may not be eliminated. In other words, some of the selected antibodies in *Pt* whose rank indicators are low may remain in the next iteration. In addition, a small selection ratio can prevent the removal of an uncontrollable large number of antibodies while providing some degree of diversity preservation. This paper set *S* =0.02.

### DMOIOB algorithm

We propose a dynamic MOIO biclustering algorithm (DMOIOB) to mine biclusters from the microarray datasets to attain the global optimum solutions. We incorporates the following three strategies: 1) ϵ-dominance to quicken convergence speed; 2) Sigma method to find good local guides; 3) population-growing strategy to increase the population size to promote exploration capability; and 4) population declining strategy to prevent the population size from growing excessively.

The pseudo-code of the proposed DMOIOB algorithm is given in Algorithm 2.

DMOIOB algorithm iteratively updates the antigens population until user-defined number of generation are generated and last converges to the optimal solution.

## Results

This paper applies the proposed DMOIOB algorithm to mine biclusters from two well known datasets and compare the diversity and convergence of the DMOIOB algorithm with MOIB algorithm. Lastly, the biological relevance of the biclusters found by DMOIOB is given.

### Datasets and data preprocessing

The first dataset is the yeast Saccharomyces cerevisiae cell cycle expression data [26], and the second dataset is the human B-cells expression data [27].

The yeast dataset collects expression level of 2,884 genes under 17 conditions. All entries are integers lying in the range of 0-600. Out of the yeast dataset there are 34 missing values. The 34 missing values are replaced by random number between 0 and 800 [1].

The human B-cells expression dataset is collection of 4,026 genes and 96 conditions, with 12.3% missing values, lying in the range of integers -750-650.The missing values are replaced by random numbers between -800-800 [1]. However, those random values affect the discovery of biclusters[28]. For providing a fair comparison with existing methods here set the same parameter for δ as [1], i.e., for the yeast data δ=300, for the human B-cells expression data δ=1200. The two gene expression dataset are taken from [1].

### Testing

DMOIOB algorithm is implemented in JAVA programming language and is performed on a 1.7GHz Pentium 4PC with 512M of RAM running Windows XP. To evaluate its performance, the proposed algorithm is compared to MOIB [15] algorithm on two well known datasets, the yeast cell cycle expression data [26] and the human B-cells expression data [27].

### Yeast dataset

Table 1 shows the information of six biclusters out of the one hundred biclusters found on the yeast dataset. The first hundred biclusters found by the proposed algorithm cover 75.8% of the genes, 100% of the conditions and in total 56.7% cells of the expression matrix. The biclusters found by MOIB [15] cover 62.4% of the genes, 100% of the conditions and in total 54.8% cells of the expression matrix. While an average coverage of 51.34% cells is reported in MOEB [7].

**Table 1:** Information of biclusters found on yeast dataset

Bicluster	Genes	Conditions	Residue	Row Variance
1	1	16	238.54	789.25
22	91	17	210.58	685.36
24	563	12	201.55	875.65
29	1233	9	275.69	896.35
78	145	13	225.11	745.65
98	874	11	207.98	874.01

Table 1 shows the number of genes and conditions, the mean squared residue and the row variance of six biclusters out of the one hundred biclusters found on the yeast dataset.

**Figure 1:**
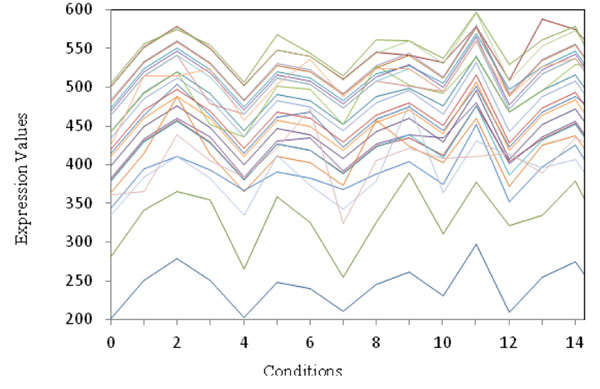
**Small biclusters of size 26×15 on the yeast dataset** Figure 1 shows the expression value of 26 genes under 15 conditions from the small biclusters(bicluster 22).

Figure 1 depicts sample gene expression profiles for small biclusters (bicluster 22) for the yeast dataset. They present a similar behaviour, and there are two genes with lower expression level than that those of the main group of genes on all the conditions.

### Human B-cells expression dataset

Table 2 shows the information of six biclusters out of the one hundred found on the human dataset. Table 2 shows that the first hundred biclusters found by the proposed algorithm cover 39.1% cells of microarray dataset (51.2%of the genes and 100% of the conditions). The one hundred biclusters found by MOIB [15] on the human dataset cover 33.6% cells of dataset(43.7%of the genes and 100% of the conditions), whereas an average of 20.96% cells are covered in MOEB [7].

**Table 2:** Biclusters found on human dataset

Bicluster	Genes	Conditions	Residue	Row Variance
1	597	49	855.69	3584.54
3	611	45	911.58	2875.12
8	1024	31	887.54	3012.25
10	478	39	812.88	6854.54
22	874	29	874.96	8740.24
31	698	37	800.74	4870.91

Table 2 shows the number of genes and conditions, the mean squared residue and the row variance of six biclusters out of the one hundred biclusters found on the human dataset.

### Comparative analysis

In this section, this paper compares the proposed algorithm with MOIB algorithm on the yeast dataset and the human dataset and the results are showed in Table 3.

**Table 3:** Comparative study of three algorithms

Algorithm	Dataset	Avg. MSR	Avg. size	Avg. time
DMOIOB	Yeast	201.86	2841.08	88.02
	Human	832.79	7106.51	258.48
MOIB	Yeast	202.32	2638.74	108.12
	Human	839.74	6918.29	280.76

Table 3 compares the performance of two algorithms. It gives the average of mean squared residue and the average size of the found biclusters, and gives computation cost of two algorithms.

From Table 3, the biclusters found by DMOIOB has a slightly higher squared residue and a higher bicluster size than those by MOIB on both yeast dataset and human dataset. It is clear from the above results that the proposed MOIB algorithm performs best in maintaining the diversity of solutions.

For computation cost we find that the computation time of MOIOB is 88.02s on yeast dataset and 258.48s on human dataset, is superior to that of MOIB.

In total it is clear from the above results that the proposed DMOIOB algorithm performs best in maintaining diversity, achieving convergence.

### Biological analysis of biclusters

We determine the biological relevance of the biclusters found by DMOIOB on the yeast dataset in terms of the statistically significant GO annotation database. The gene ontology (GO) project (http://www.geneontology.org) provides three structured, controlled vocabularies that describe gene products in terms of their associated biological processes, cellular components and molecular functions in a species-independent manner. To better understand the mining results, we feed genes in each bicluster to Onto-Express(http://vortex.cs.wayne.edu/Projects.html) and obtain a hierarchy of functional annotations in terms of Gene Ontology for each bicluster.

The degree of enrichment is measured by p-values which use a cumulative hyper-geometric distribution to compute the probability of observing the number of genes from a particular GO category (function, process and component)within each bicluster. The p-values are calculated for each functional category in each bicluster to denote how well those genes match with the corresponding GO category given in Table 4.

**Table 4:** Significant GO terms of genes in three biclusters

Cluster No.	No. of genes	Process	Function	Component
1	99	Response to DNA damage stimulus (n=21,p=0.0016)	RNA polymerase II transcription factor activity (n=11,p=0.0064)	Intracellular membrane-bound organelle (n=16,p=0.0025)
22	91	Physiological process (n=23,p=0.0014)	MAP kinase activity (n=6,p=0.0023)	Cytosolic ribosome (n=17,p=0.0042)
78	145	Protein biosynthesis (n=52,p=0.0024)	Protein transporter activity (n=9,p=0.0021)	Cytosolic ribosome (n=12,p=0.0032)

Table 4 lists the significant shared GO terms which are used to describe genes in each bicluster for the process, function and component ontology.

## Conclusions

This paper has provided a novel dynamic multi-objective immune optimization biclustering framework for mining biclusters from microarray datasets. We focus on finding maximum biclusters with lower mean squared residue and higher row variance. Those three objectives are incorporated into the framework with three fitness functions. We apply immune clonal selection principle and Sigma method to find better local guide in objective space and combine ε-dominance and crowding distance strategy to improve the diversity of the solutions and to quicken convergence of the algorithm; a population adding method that dynamically grows new individuals with enhanced exploration and exploitation capabilities; a population decreasing strategy to balance and control the dynamic population size. The results on the yeast microarray dataset and the human B-cells expression dataset verify the good quality of the found biclusters, and comparative analysis show that the proposed MOIB is superior to MOIB algorithm in terms of the diversity of solutions and the convergence of the algorithm.

## Competing interests

The authors declare that they have no competing interests.

## Authors' contributions

JL design DMOBIO to mine biclusters from gene expression data and drafted the manuscript. ZL, XH and EP were involved in study design and coordination and revised the manuscript. YC conducted the algorithm design.
